# Loss of COPZ1 induces NCOA4 mediated autophagy and ferroptosis in glioblastoma cell lines

**DOI:** 10.1038/s41388-020-01622-3

**Published:** 2021-01-08

**Authors:** Yulin Zhang, Yang Kong, Yuan Ma, Shilei Ni, Tobias Wikerholmen, Kaiyan Xi, Feihu Zhao, Zhimin Zhao, Junpeng Wang, Bin Huang, Anjing Chen, Zhong Yao, Mingzhi Han, Zichao Feng, Yaotian Hu, Frits Thorsen, Jian Wang, Xingang Li

**Affiliations:** 1 Department of Neurosurgery, Qilu Hospital and Institute of Brain and Brain-Inspired Science, Cheeloo College of Medicine, Shandong University, Shandong 107 Wenhua Xi Road, Jinan, 250012 P.R. China; 2 Shandong Key Laboratory of Brain Function Remodeling, Shandong 107 Wenhua Xi Road, Jinan, 250012 P.R. China; 3 Department of Biomedicine, University of Bergen, Jonas Lies vei 91, 5009 Bergen, Norway; 4 Molecular Imaging Center, Department of Biomedicine, University of Bergen, Jonas Lies vei 91, 5009 Bergen, Norway

**Keywords:** Cancer metabolism, Cancer therapy, CNS cancer

## Abstract

Dysregulated iron metabolism is a hallmark of many cancers, including glioblastoma (GBM). However, its role in tumor progression remains unclear. Herein, we identified coatomer protein complex subunit zeta 1 (COPZ1) as a therapeutic target candidate which significantly dysregulated iron metabolism in GBM cells. Overexpression of *COPZ1* was associated with increasing tumor grade and poor prognosis in glioma patients based on analysis of expression data from the publicly available database The Cancer Genome Atlas (*P* < 0.001). Protein levels of COPZ1 were significantly increased in GBM compared to non-neoplastic brain tissue samples in immunohistochemistry and western blot analysis. SiRNA knockdown of COPZ1 suppressed proliferation of U87MG, U251 and P3#GBM in vitro. Stable expression of a COPZ1 shRNA construct in U87MG inhibited tumor growth in vivo by ~60% relative to controls at day 21 after implantation (*P* < 0.001). Kaplan–Meier analysis of the survival data demonstrated that the overall survival of tumor bearing animals increased from 20.8 days (control) to 27.8 days (knockdown, *P* < 0.05). COPZ1 knockdown also led to the increase in nuclear receptor coactivator 4 (NCOA4), resulting in the degradation of ferritin, and a subsequent increase in the intracellular levels of ferrous iron and ultimately ferroptosis. These data demonstrate that COPZ1 is a critical mediator in iron metabolism. The COPZ1/NCOA4/FTH1 axis is therefore a novel therapeutic target for the treatment of human GBM.

## Introduction

Glioblastoma (GBM) is the most common primary malignant brain tumor in adults, with an annual incidence of 5.26 per 100,000 population [1, 2]. Prognosis and the quality of life of GBM patients are poor [3]. Median survival of patients is around 14 months, despite aggressive treatment including surgery, radiotherapy, and chemotherapy [4]. Compared to normal cells, GBM cells have altered expression of many iron metabolism-related proteins and iron-related enzyme activities [5]. These changes often contribute to the relatively high availability of iron in GBM cells and promote the function of iron-dependent proteins that are involved in many physiological processes, such as tumor initiation, progression, and metastasis [6–8]. Targeting iron-related proteins or increasing intracellular iron levels are considered to be feasible strategies for treating cancers [9, 10]. Our recently published work also found that inducing ferroptosis in GBM can achieve good therapeutic effects [11]. However, an understanding of the molecular mechanisms involved in the process in GBM will help to exploit ferroptosis in the treatment of the disease.

Ferroptosis is an iron-dependent form of regulated cell death which occurs as a consequence of lethal lipid peroxidation [12]. Although the physiological function of ferroptosis is not clearly defined, the accumulation of reactive oxygen species (ROS) exceeding the capacity of glutathione (GSH) has been shown to induce ferroptosis [13]. Iron is an essential cofactor of metabolic enzymes, and is tightly integrated with many biological processes such as neurotransmitter transmission, oxygen transport, cellular division and energy generation [14]. Disruption of normal iron transport may cause a build up of iron within the cells, drive intracellular ROS production by the Fenton reaction, which is a catalytic process that converts ferrous iron and hydrogen peroxide into a highly toxic free radical, triggering lipid peroxidation, which has cytotoxic effects [15, 16]. Ferroptosis occurs in several human diseases, such as ischemic heart diseases, brain damage, kidney failure and cancer [17–20]. However, very little is currently known about the role of ferroptosis in GBM.

Several proteins have been shown to be involved in ferroptosis, including iron chaperones poly (rC)-binding protein 1 (PCBP1) [21], nuclear receptor coactivator 4 (NCOA4) [22], iron-responsive element-binding protein 2 [23], and heat shock protein beta-1 [24]. Coatomer protein complex subunit zeta 1 (COPZ1), which belongs to the coatomer protein complex I, is involved in intracellular trafficking, endosome maturation, lipid homeostasis, and autophagy [25, 26]. Intriguingly, COPZ1 is associated with iron metabolism through regulation of transferrin (TF) [27]. It is also involved in the homeostasis of hepcidin, a key regulator of iron entry into mammalian blood circulation [28].

In thyroid tumor cells, depletion of COPZ1 leads to cell death, suggesting it has potential as a therapeutic target for thyroid cancer, furthermore, subcutaneous xenograft models locally injected with siRNAs against COPZ1 reduced thyroid tumor growth [29]. In this study, we provide the first evidence that decreased COPZ1 expression induces ferroptosis, and that it is mediated by the NCOA4 protein in human GBM cells. These discoveries not only identify a novel role for COPZ1 in ferroptosis, but also validate manipulating the ferroptotic process as a potential therapeutic strategy in the treatment of GBM patients.

## Results

### *COPZ1* is overexpressed in human gliomas and predicts poor prognosis

**Fig. 1:**
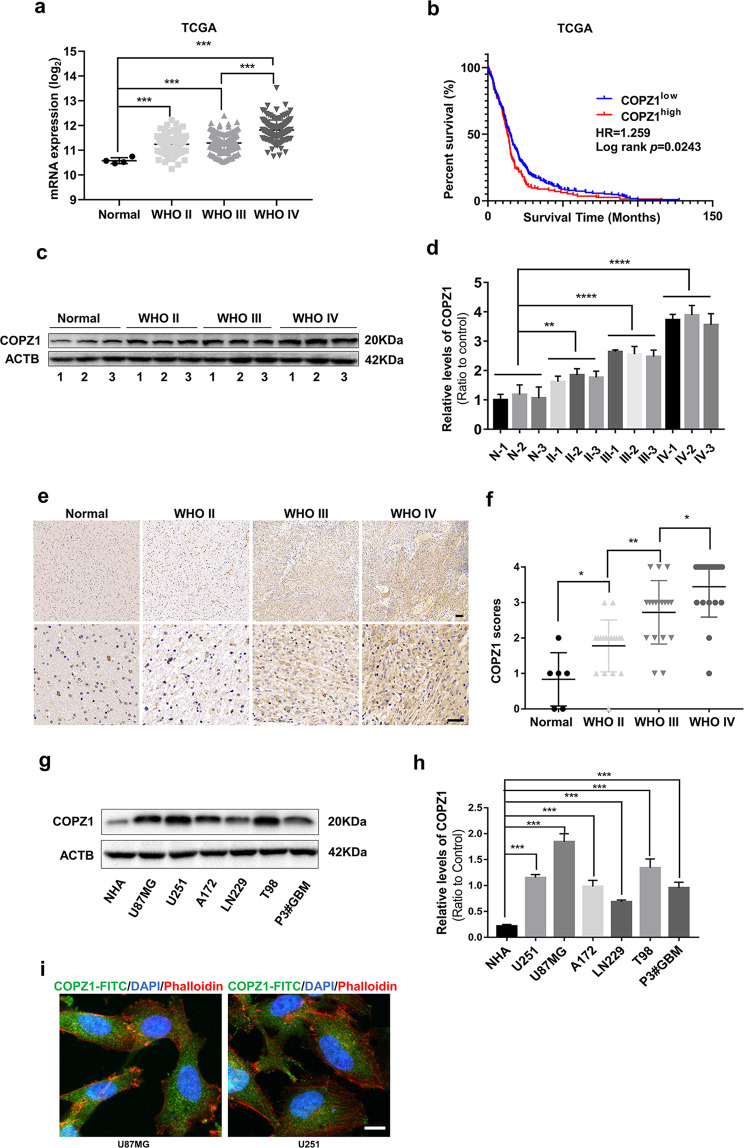
COPZ1 expression is elevated in primary human glioma samples and GBM cell lines. **a** *COPZ1* RNA expression (log_2_) based on the 2016 WHO classification from the TCGA database. **b** Kaplan–Meier survival analysis of patient overall survival data based on high versus low expression of *COPZ1* from the TCGA dataset. **c** Representative western blot of protein levels of COPZ1 in lysates (20 µg) prepared from human glioma tissues (*n* = 9) and non-neoplastic brain tissues (*n* = 3). **d** Relative quantification of western blots shown in (**c**). **e** Representative images of IHC staining for COPZ1 in human glioma and non-neoplastic brain tissue samples (Normal, *n* = 6; WHO II, *n* = 18; WHO III, *n* = 18; WHO IV, *n* = 18). Scale bar for the upper images, 50 µm and the lower images, 100 µm. **f** Graphical results showing the IHC scores. **g** Representative western blot of COPZ1 protein levels in normal human astrocytes (NHA) and human GBM cell lines. **h** Relative quantification of western blots shown in (**g**). **i** Immunofluorescence staining of COPZ1 (green) in U87MG and U251 cells analyzed with fluorescence microscopy. The nuclei were stained with DAPI (blue), while the cytoskeleton was stained with pholloidin (red). Scale bar, 10 µm. One-way ANOVA for multi-group comparisons: *p < 0.05, **p < 0.01, ***p < 0.001; log-rank test: *p* < 0.05.

To begin to examine the role of COPZ1 in the development of human glioma, we first examined mRNA levels of the gene in human glioma samples using the expression data in the publicly available dataset from The Cancer Genome Atlas (TCGA). *COPZ1* mRNA levels were increased in low grade (WHO II; *n* = 226) and high-grade gliomas (WHO III, *n* = 244; WHO IV, *n* = 150; *p* < 0.0001) relative to non-neoplastic brain tissue samples (*n* = 4) (Fig. 1a). Kaplan–Meier analysis of the TCGA dataset also demonstrated that high *COPZ1* expression in tumors (>the median value) predicted shorter overall survival in patients (Fig. 1b). Analysis of the publicly available Rembrandt dataset yielded similar results (Supplementary Fig. 1a, b). We found a corresponding increase in COPZ1 protein levels in western blot analysis of lysates prepared from primary human glioma specimens relative to non-neoplastic brain tissue samples (~4×, grade IV vs non-neoplastic tissue samples) (Fig. 1c, d). Immunohistochemical (IHC) staining performed on 60 paraffin-embedded clinical samples, including grade II (*n* = 18), grade III (*n* = 18), grade IV (*n* = 18) and non-neoplastic brain tissue samples (*n* = 6), confirmed the result that COPZ1 increased with increasing tumor grade. (Fig. 1e, f). Furthermore, we examined other factors such as age, gender, tumor size, liquefactive necrosis, preoperative tumor edema, and tumor grade. The results showed that COPZ1 expression was positively associated with tumor grade and liquefactive necrosis, independent from age, gender, tumor size, and edema, which suggested that COPZ1 could be a potential diagnostic marker for glioma patients (Table 1, *p* < 0.05).

**Table 1:** Correlations of COPZ1 expression with clinicopathological features in glioma patients.

Variables	*n*	COPZ1 expression		*P* value
		Low	High	
Age (year)				
<60	37	18	19	0.359
≥60	17	6	11	
Gender				
Male	29	12	17	0.625
Female	25	12	13	
Tumor size (cm)				
<4	18	9	9	0.561
≥4	36	15	21	
Liquefactive necrosis				
Negative	35	19	16	0.048
Positive	19	5	14	
Edema				
None to mild	16	9	7	0.257
Moderate to severe	38	15	23	
WHO grade				
II	18	16	2	<0.001
III	18	6	12	
IV	18	2	16	

Finally, western blot analysis demonstrated that COPZ1 protein levels were also elevated in human glioma cells U87MG, U251, A172, LN229, T98 and P3#GBM relative to normal human astrocytes (NHA) in culture (Fig. 1g, h). Immunofluorescence staining showed that COPZ1 was mainly localized within the cytoplasm of U87MG and U251 cells (Fig. 1i). Taken together, these results indicate that COPZ1 may have an important role in glioma progression and serve as a novel diagnostic marker.

### Silencing COPZ1 inhibits glioma cell proliferation and induces cell death

**Fig. 2:**
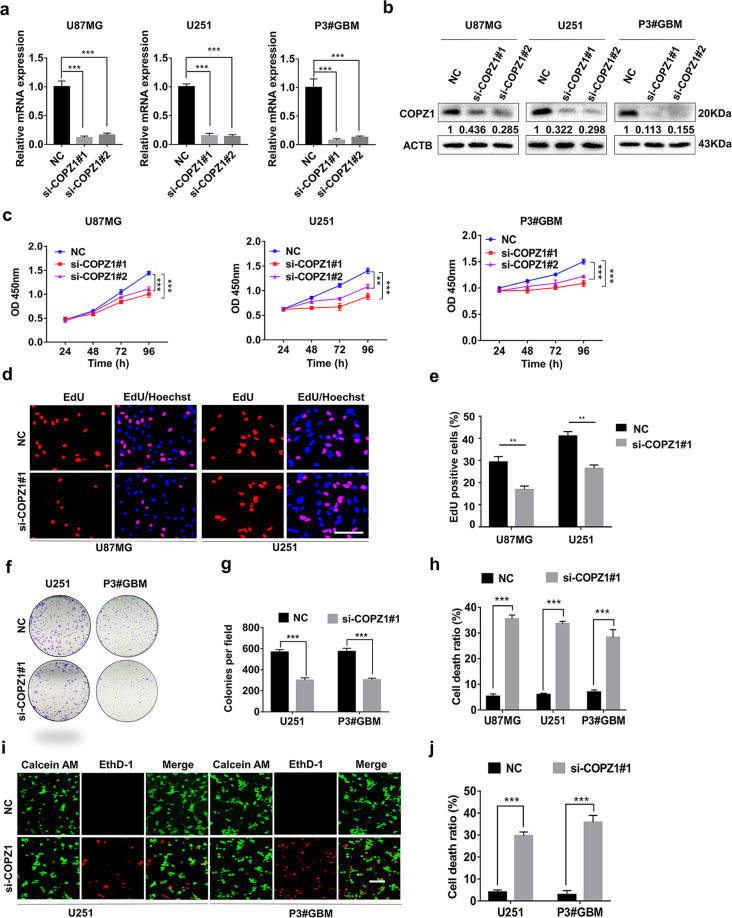
Silencing of COPZ1 inhibits GBM cell viability and proliferation. **a** qRT-PCR to detect *COPZ1* mRNA levels in U87MG, U251, and P3#GBM cells transfected with two independent COPZ1 siRNAs, si-COPZ1#1, and si-COPZ1#2. **b** Western blot analysis of COPZ1 protein levels in U87MG, U251, and P3#GBM cells transfected with si-COPZ1#1 and si-COPZ1#2. **c** Growth curves for si-COPZ1 transfected U87MG, U251, and P3#GBM cells generated with OD 450 readings plotted over time using the CCK8 assay. **d** Fluorescence images of EdU assays performed on U87MG and U251 cells transfected with si-COPZ1#1. Nuclei were stained with DAPI (blue). Scale bar, 100 μm. **e** Graphic representation of the ratios of EdU positive cells in U87MG and U251 cells transfected with si-COPZ1#1. **f** Representative images of colony forming assays for U251 and P3#GBM cells transfected with si-COPZ1#1 to evaluate cell proliferation. Cells were fixed and stained with crystal violet, and colonies were counted. **g** Graphic representation of the number of colonies shown in (**f**). **h** LDH release assay for si-COPZ1#1 transfected U87MG, U251, and P3#GBM cells compared to their respective control cells. **i** Representative images of live (green)/dead (red) assays for U251 and P3#GBM cells transfected with si-COPZ1#1. Scale bar, 100 μm. **j** Graphic representation of the rate of dead (red) cells in U251 and P3#GBM cells transfected with si-COPZ1#1. Student’s *t* test for two-group comparison: *p < 0.05, **p < 0.01, ***p < 0.001; one-way ANOVA for multi-group comparisons: *p < 0.05, **p < 0.01, ***p < 0.001.

We examined the biological effect of knocking down COPZ1 with two small interfering RNAs (si-COPZ1#1 and si-COPZ1#2). Knockdown with either siRNA reduced COPZ1 mRNA and protein levels by ~80 and 60%, respectively (Fig. 2a, b). Likewise, cell growth was significantly inhibited in si-COPZ1 compared to NC cells (Fig. 2c). In EdU (Fig. 2d, e) and colony formation assays (Fig. 2f, g), cell proliferation of U251 and P3#GBM cells transfected with si-COPZ1#1 was also reduced. Stable knockdown was also achieved by infecting cells with lentiviral constructs expressing two different shRNAs (Supplementary Fig. 2a).

To examine mechanisms mediating inhibition of cell growth in COPZ1 knockdown, we assessed cell death using LDH release and the Live/Dead viability assay. The release of LDH increased in U87MG, U251, and P3#GBM cells by ~30% with loss of COPZ1 (Fig. 2h). In the Live/Dead cell viability assay, the number of dead U251 and P3#GBM cells transfected with si-COPZ1#1 (red fluorescence) was elevated to 29.6% and 35.8% compared to the control (4.0% of U251cells and 2.8% of P3#GBM cells, Fig. 2i, j). Collectively, these data suggest that COPZ1 knockdown inhibits cell proliferation and induces cell death in GBM cell lines in culture.

### Knockdown of COPZ1 increases intracellular iron levels

**Fig. 3:**
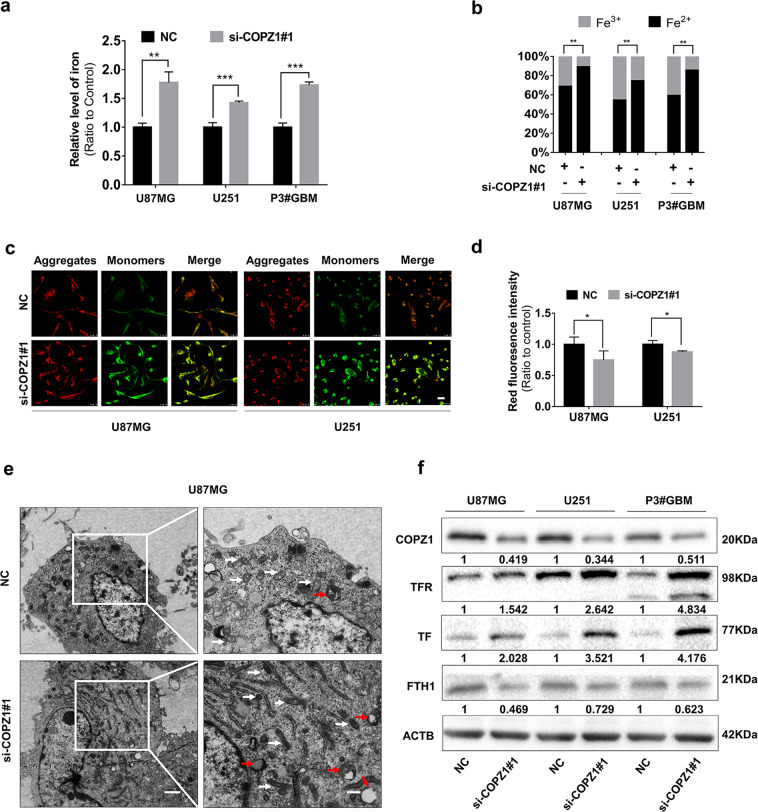
COPZ1 knockdown induces changes in iron metabolism. **a** Total intracellular iron in U87MG, U251 and P3#GBM glioma cells after transfection with si-COPZ1#1 at 24 h. **b** Graphic representation of the levels of intracellular ferrous iron compared to ferric iron, as evaluated with the colorimetric iron assay kit. **c** Representative fluorescence images of U87MG and U251 cells stained with the JC-1 probe to assess mitochondrial membrane potential. Scale bar, 25 μm. **d** Statistical analysis of the red and green fluorescence shown in (**c**). **e** Images from transmission electron microscopy showing morphology of mitochondria (white arrows) and formation of autophagosomes (red arrows) in U87MG cells transfected with si-COPZ1#1 for 24 h. Mitochondria show increased membrane density (white arrows) and a shrunken morphology. Scale bar,1.2 μm (left) and 0.6 μm (right). **f** Western blot analysis of TFR, TF, and FTH1 in lysates prepared from U87MG, U251, and P3#GBM cells transfected with si-COPZ1#1. Student’s *t* test: *p < 0.05, **p < 0.01, ***p < 0.001.

Since COPZ1 is involved in iron metabolism [27, 30], we next studied whether knockdown of COPZ1 induced ferroptosis in GBM cells. First, we assessed intracellular iron levels in si-COPZ1#1 transfected U87MG, U251 and P3#GBM cells compared to controls. With loss of COPZ1, the intracellular iron levels were increased by ~ 70% (Fig. 3a). The proportion of ferrous iron levels (Fe^2+^) was also increased relative to ferric iron (Fe^3+^, Fig. 3b), indicating that intracellular ferrous iron was upregulated with knockdown of COPZ1.

Increased intracellular iron induces ferroptosis, and a hallmark of ferroptosis is morphological changes in mitochondria [17]. Therefore, we used the ratio of red to green fluorescence of JC-1 dye in cells to detect possible changes in mitochondrial membrane potential. JC-1 aggregates at high concentrations and emits red fluorescence in normal mitochondria but exists as a green fluorescing monomer at lower concentrations under depolarization. The ratio of red to green fluorescence in U87MG cells transfected with si-COPZ1#1 decreased, indicating a reduction in the mitochondrial membrane potential as less dye aggregated in the organelles (Fig. 3c, d). These results were consistent with the morphology of mitochondria in transfected cells characterized with transmission electron microscopy. U87MG cells with loss of COPZ1 displayed shrunken mitochondria with increased membrane density (Fig. 3e).

To determine the mechanism underlying the increase in intracellular iron, we performed western blot analysis to detect levels of two proteins involved in the uptake of iron, TF and the transferrin receptor (TFR). TF binds to iron and the TFR transfers the TF-iron complex into cells. Levels of both TF and TFR were increased in si-COPZ1#1 transfected U87MG, U251, and P3#GBM cells. Furthermore, ferritin (FTH1), which regulates intracellular ferrous iron, was down-regulated (Fig. 3f). Fluorescence staining of TFR validated these results, demonstrating that the protein was increased in cells with COPZ1 knockdown (Supplementary Fig. 3a, 3b). We next performed western blots to investigate the role of the FBXL5/IRP2 pathway, which plays a vital function in iron metabolism, in GBM. The western blot results showed no significant alterations in the levels of FBXL5 nor IRP2 in our glioma cell lines (Supplementary Fig. 3c). Our results are therefore consistent with the hypothesis that knockdown of COPZ1 induces iron accumulation by promoting cellular uptake of iron.

### Knockdown of COPZ1 causes ferroptosis in GBM cells

**Fig. 4:**
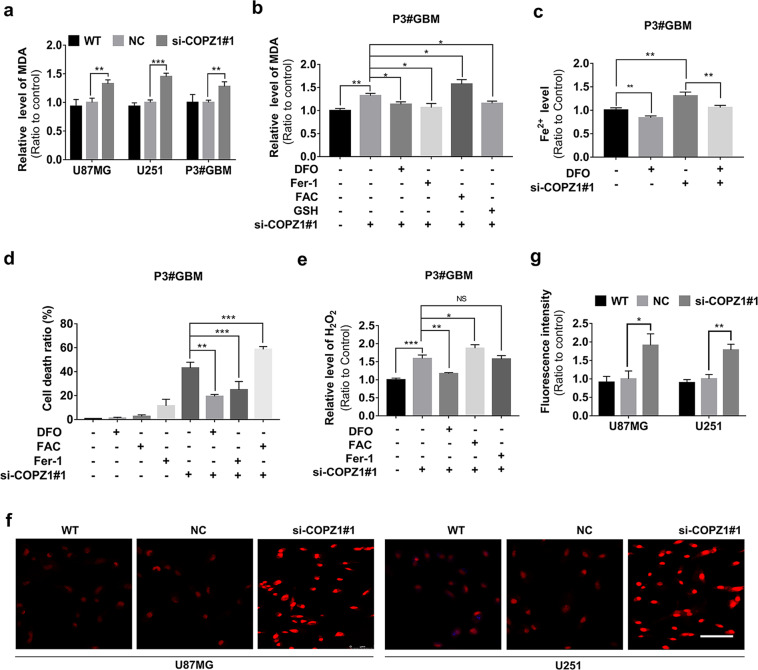
Intracellular iron increases with loss of COPZ1 and enhances cell death by causing ROS and lipid peroxidation. **a** MDA levels detected in U87MG, U251 and P3#GBM glioma cells transfected with si-COPZ1# for 48 h. **b** MDA levels detected in si-COPZ1#1 transfected cells pretreated with DFO, Fer-1, FAC, or GSH. **c** Iron assay to detect ferrous iron levels in the presence of DFO. **d** LDH release assay of transfected cells treated with DFO, Fer-1, or FAC. **e** Hydrogen peroxide assay showing accumulation of H_2_O_2_ in P3#GBM cells transfected with si-COPZ1#1 and treated with DFO, FAC and Fer-1 relative to controls. **f** Representative images of dihydroethidium (DHE; red fluorescence) superoxide probe 48 h after transfection of cells with si-COPZ1#1. For each group of 2 cell lines, 3 images from triplicate experiments were counted. Scale bar, 75 μm. **g** Statistical analysis of the fluorescence intensities of U87MG and the U251 GBM cell lines transfected with si-COPZ1#1 compared to their respective control cells. Student’s *t* test for two-group comparison: *p < 0.05, **p < 0.01, ***p < 0.001; one-way ANOVA for multi-group comparisons: NS = non-significant, *p < 0.05, **p < 0.01, ***p < 0.001.

Ferroptosis is characterized by lipid peroxidation, and the final product of lipid peroxidation is malondialdehyde (MDA) [12, 31]. Thus, we examined whether loss of COPZ1 led to changes in MDA levels. MDA levels were significantly increased in si-COPZ1#1 transfected U87MG, U251, and P3#GBM cells compared to controls (Fig. 4a). BODIPY 581/591 staining confirmed these results (Supplementary Fig. 4a). To test whether the increase in MDA was an iron dependent process, transfected cells were exposed to the iron chelator deferoxamine (DFO). DFO suppressed the increase of MDA in si-COPZ1#1 transfected cells (Fig. 4b, Supplementary Fig. 4b, 4c). Cells pretreated with DFO (600 µmol/L) also showed reduced levels of intracellular ferrous iron (Fig. 4c, Supplementary Fig. 4d, 4e). However, increasing intracellular iron through exposure to ferric ammonium citrate (FAC) led to further increases in MDA levels in si-COPZ1#1 transfected cells (Fig. 4b, Supplementary Fig. 4b, c). The increase in MDA was also inhibited in transfected P3#GBM cells pretreated with GSH, the critical tripeptide antioxidant (Fig. 4b). These results indicated that the increase in intracellular iron was due to loss of COPZ1 induced lipid peroxidation.

To determine whether increased MDA in si-COPZ1#1 transfected cells was associated with cell death, cells were treated with ferrostatin-1 (Fer-1, 50 µmol/L), a small molecule scavenger of free radical species involved in lipid peroxidation. Both the levels of MDA and the cell death rate were decreased in si-COPZ1#1 transfected cells treated with Fer-1 (Fig. 4b, d). Increasing iron in cells through exposure to FAC also led to increased cell death. However, pretreatment with DFO alleviated cell death (Fig. 4d, Supplementary Fig. 4f, 4g). To better understand the role of ferroptosis in cells with loss of COPZ1, apoptosis and necrosis inhibitors were used to examine their contribution to cell death in our model (Supplementary Fig. 4h). Interestingly, we found that ferroptosis is not the sole process of cell death in our model, with necrosis and apoptosis contributing to cell death by 14.5% and 7.7%, respectively. Thus, the observed effect of COPZ1 knockdown on cell viability is mainly due to dysregulation of iron metabolism sensitizing cells to ferroptosis, and partly attributable to necrosis and apoptosis. In summary, these results suggest that lipid peroxidation may contribute to cell death induced in GBM cell lines through the loss of COPZ1.

### Elevated intracellular iron levels increase the production of reactive oxygen species

ROS, such as hydrogen peroxide (H_2_O_2_), superoxide radicals (O_2_^−^) and the highly cytotoxic hydroxyl radicals (•OH) are the main causes of intracellular oxidative stress [32, 33]. Increases in iron can trigger the Fenton reaction which converts hydrogen peroxide (in the presence of ferrous iron) into superoxide radicals and hydroxyl radicals and releases ferric iron, these hydroxyl radicals cause lipid peroxidation [34]. Therefore, we examined whether loss of COPZ1 altered intracellular levels of H_2_O_2_. H_2_O_2_ accumulated to a greater level in cells transfected with si-COPZ1#1 knockdown compared to the controls (Fig. 4e). In addition, the increase in H_2_O_2_ was enhanced with FAC and blocked with DFO (Fig. 4e). As H_2_O_2_ is produced from superoxide, we therefore investigated the generation of superoxide using the red fluorescent superoxide probe, dihydroethidium (DHE). Fluorescence intensities detected in si-COPZ1#1 transfected cells were significantly greater than in controls (Fig. 4f, g).

The small molecule erastin induces ferroptosis by inhibiting system X_c_^-^, which leads to lipid peroxidation. In both WT and si-COPZ1 transfected cells, erastin promoted cell death and enhanced MDA levels. (Supplementary Fig. 4i, 4j). Fer-1, however, not only reversed the effect of erastin treatment on cell death and MDA levels in WT and si-COPZ1 transfected cells, but it also seemed to reverse the effect of the si-COPZ treatment. In conclusion, increased iron levels due to the loss of COPZ1 led to elevated production of intracellular H_2_O_2_ and superoxide.

### COPZ1 depletion induces autophagy in GBM cells in vitro

**Fig. 5:**
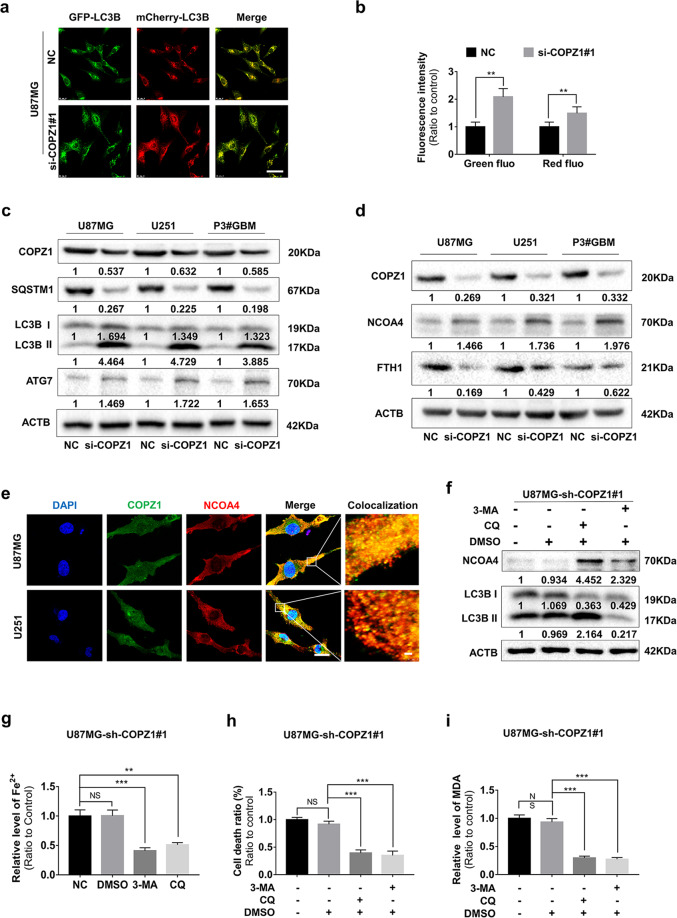
Loss of COPZ1 induces autophagy in GBM cells in vitro. **a** Representative fluorescence images of GFP/mCherry-LC3B puncta in si-COPZ1#1 transfected U87MG after 48 h. Scale bars, 25 μm. **b** Statistical analysis of the images shown in (**a**). Quantification represents results taken from 3 images from experiments performed in triplicate. **c** Representative western blots showing protein levels of LC3B, SQSTM1 (p62), ATG7 and ACTB (loading control) in si-COPZ1#1 transfected U87MG, U251, and P3#GBM cells. **d** Western blots showing protein levels of FTH1, NCOA4, and ACTB (loading control) in si-COPZ1#1 transfected U87MG, U251, and P3#GBM cells. **e** Representative fluorescence images showing intracellular localization of COPZ1 (green) and NCOA4 (red). Alexa-NCOA4 was diffusely localized in the cytoplasm and largely colocalized with FITC-COPZ1 in normal U87MG and U251 cells. Scale bar, 10 μm; scale bars of magnified images, 1 μm. **f** Western blots to detect levels of NCOA4 and LC3B in U87MG-sh-COPZ1#1 cells after pretreatment with 3-MA (10 mM) and CQ (3 μM) for 1 h. Data are representative of 3 independent experiments. **g** Relative iron levels in U87MG-sh-COPZ1#1 cells after pretreatment with 3-MA (10 mM) and CQ (3 μM) for 1 h. **h** Cell death ratio after pretreatment with 3-MA (10 mM) and CQ (3 μM) for 1 h in U87MG-sh-COPZ1#1 cells. **i** MDA levels after pretreatment of U87MG-sh-COPZ1#1 cells with 3-MA (10 mM) and CQ (3 μM) for 1 h. Student’s *t* test for two-group comparison: **p < 0.01; one-way ANOVA for multi-group comparisons: NS = non-significant, **p < 0.01, ***p < 0.001.

Autophagy is a conserved degradation pathway maintaining intracellular homeostasis [35]. However, excessive autophagy will promote cell death rather than contribute to cell survival [36]. COPZ1 depletion has also been shown to induce lethal autophagy in tumor cells [29, 37]. TEM also revealed an increase in the number of autophagosomes in si-COPZ1# transfected U87MG cells (Fig. 3e). Therefore, we determined whether loss of COPZ1 induced autophagy in GBM cells. Autophagy occurs in different stages. The progression from formation of autophagosomes to degradation of proteins is detected with the pH sensitive tandem fusion of GFP/mCherry to the protein LC3, which is involved in the formation of autophagosomes. Co-localization of green (GFP) and red fluorescence (mCherry) occurs in the formation of autophagosomes. U87MG cells were transfected first with a lentivirus expressing GFP/mCherry-LC3 for 48 h and then si-COPZ1#1 for 24 h. Under confocal microscopy, the GFP/mCherry-LC3 puncta increased in the si-COPZ1#1 transfected cells, and the green and red fluorescence intensities were elevated ~ 2× and 1.6× compared to the NC group (Fig. 5a, b).

Protein markers of autophagosomes and autophagy were also examined using western blot analysis of lysates prepared from si-COPZ1#1 transfected cells. A protein involved in the formation of autophagosomes, LC3B-II, was increased in si-COPZ1#1 transfected U87MG, U251, and P3#GBM cells (Fig. 5c). Autophagy-related protein 7 (ATG7), which plays a central role in mediating autophagy, was also increased in the si-COPZ1#1 transfected cells (Fig. 5c). In contrast, levels of a protein substrate in autophagy, SQSTM1 (P62), were decreased (Fig. 5c). Taken together, these results indicate that loss of COPZ1 promoted autophagy in GBM cells in culture.

### NCOA4 plays a central role in autophagy induced by the loss of COPZ1 in GBM cells

Ferritin is the main iron storage protein complex in cells, and it consists of FTL1 (ferritin light polypeptide 1) and FTH1 (ferritin heavy polypeptide 1) [38]. Recent studies have shown that increased autophagy promotes the degradation of ferritin and increases intracellular iron content, leading to the Fenton reaction and subsequent ferroptosis [22]. On western blot, FTH1 levels were decreased in si-COPZ1#1 transfected U87MG, U251, and P3#GBM cells (Fig. 5d).

It is well-known that NCOA4 is a selective cargo receptor for the autophagic degradation of ferritin which is known as ferritinophagy [39, 40]. We therefore analyzed whether NCOA4 was involved in ferroptosis induced by the loss of COPZ1. Protein levels of NCOA4 were increased in si-COPZ1#1 transfected cells (Fig. 5d). Using immunofluorescence, we found that NCOA4 (red) was in the cytoplasm in normal U87MG and U251 cells and that it colocalized with COPZ1 (green) positive puncta (Fig. 5e).

To examine autophagic flux, U87MG-sh-COPZ1#1 cells were treated with 3-MA or chloroquine (CQ) for 48 h, and the levels of LC3B-I and LC3B-II, markers of autophagosome assembly, were examined on western blot. Treatment with 3-MA, which prevents autophagosome assembly, inhibited conversion of LC3B-I to LC3B-II. However, treatment with 3 μM CQ for 48 h, which interferes with the progression of autophagy through inhibition of the fusion of autophagosomes and lysosomes, resulted in accumulation of LC3B-II (Fig. 5f).

NCOA4 levels were increased when cells were treated with 3-MA or CQ to block autophagosome assembly or function and thus degradation (Fig. 5f, Supplementary Fig. 5a, 5b). Inhibition of ferritinophagy with 3-MA or CQ led to decreased ferrous iron levels (Fig. 5g, Supplementary Fig. 5c, 5d) and cell death ratios (Fig. 5h, Supplementary Fig. 5e, 5f). MDA levels also did not increase (Fig. 5i, Supplementary Fig. 5g, 5h). Infection of previously COPZ1 silenced U87MG cells with a lentiviral construct expressing COPZ1 (OE-COPZ1) resulted in lower expression of NCOA4 (Supplementary Fig. 5i). Transfection of si-COPZ1 into cells overexpressing NCOA4 did not change NCOA4 levels significantly compared to controls, when NCOA4 overexpressing cells were transfected with OE-COPZ1, the NCOA4 levels were inhibited (Supplementary Fig. 5j, 5k). Taken together, these results indicated that COPZ1 might negatively regulate NCOA4 activity, as knockdown of COPZ1 induces NCOA4-mediated ferritinophagy. Thus, NCOA4-mediated ferritin degradation is critical to ferroptosis induced by COPZ1 deficiency.

### NCOA4 mediated degradation of ferritin enhances ferroptosis in COPZ1 deficient cells

**Fig. 6:**
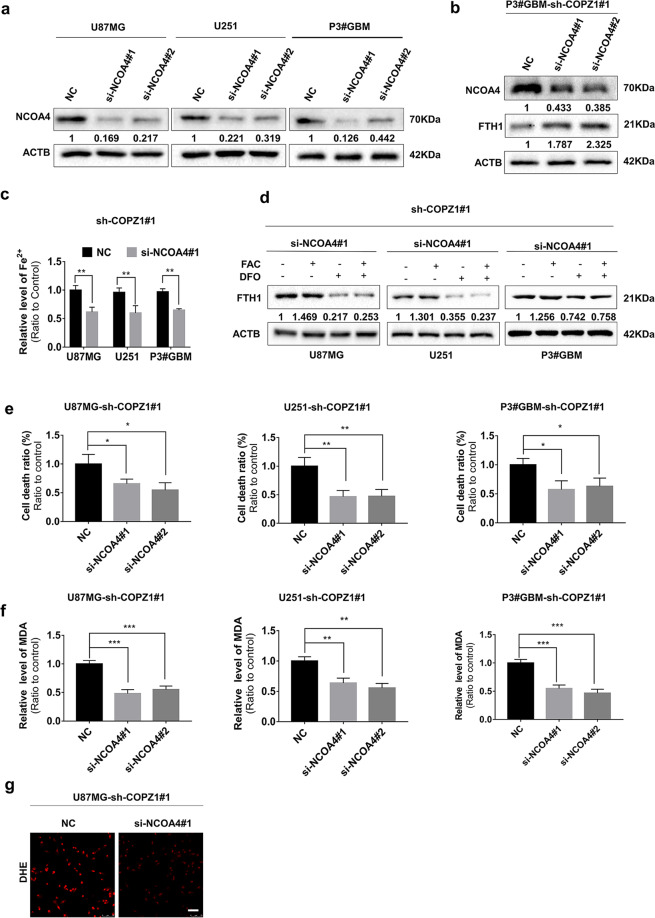
NCOA4 mediates autophagic delivery of ferritin and controls iron homeostasis. **a** Western blots showing levels of NCOA4 and ACTB (loading control) in U87MG, U251, and P3#GBM glioma cells transfected with si-NCOA4#1 and si-NCOA4#2 for 48 h. **b** Western blots showing levels of NCOA4, FTH1, and ACTB (loading control) in P3#GBM-sh-COPZ1#1 cells transfected with 2 independent siRNAs against NCOA4 (si-NCOA4#1 and si-NCOA4#2). **c** Ferrous iron levels detected after knockdown of NCOA4 in U87MG-, U251- and P3#GBM-sh-COPZ1#1 cells. **d** Western blots to detect FTH1 and ACTB (loading control) in U87MG-, U251-, and P3#GBM-sh-COPZ1#1 cells transfected with si-NCOA4#1. **e** LDH assay as a measure of cell death in U87MG-, U251-, and P3#GBM-sh-COPZ1#1 cells transfected with NCOA4 siRNAs. **f** MDA levels in U87MG-, U251-, and P3#GBM-sh-COPZ1#1 cells transfected with NCOA4 siRNAs. **g** DHE levels in U87MG-sh-COPZ1#1cells transfected with si-NCOA4#1. Student’s *t* test for two-group comparison: **p < 0.01; one-way ANOVA for multi-group comparisons: *p < 0.05, **p < 0.01, ***p < 0.001.

Since knockdown of COPZ1 elevated the levels of NCOA4 leading to degradation of ferritin, we next examined the effects of NCOA4 knockdown on ferroptosis in GBM cells. Protein levels of NCOA4 were markedly decreased on western blot in U87MG, U251 and P3#GBM cells transfected with two independent NCOA4 siRNAs (Fig. 6a). In P3#GBM-sh-COPZ1#1 cells transfected with the NCOA4 siRNAs, basal levels of ferritin levels (FTH1) were increased (Fig. 6b). This result suggests that NCOA4-deficiency counteracts the up-regulation of iron-induced by COPZ1 deficiency. Therefore, we examined the levels of ferrous iron (Fe^2+^) in U87MG-, U251- and P3#GBM-sh-COPZ1#1 cells transfected with NCOA4 siRNAs compared to controls. With knockdown of NCOA4, ferrous iron levels were decreased in U87MG-, U251-, and P3#GBM-sh-COPZ1#1 cells (Fig. 6c). Furthermore, FTH1 levels decreased with DFO despite the absence of COPZ1 (Fig. 6d), and FAC treatment did not change FTH1 levels significantly, indicating that iron metabolism might be regulated through the NCOA4-FTH1 pathway. Cell death ratios and MDA levels also decreased in the COPZ1 deficient GBM cell populations with NCOA4 knockdown (Fig. 6e, f). Finally, levels of superoxide in U87MG-sh-COPZ1#1 cells decreased with NCOA4 knockdown as assessed using DHE fluorescence (Fig. 6g).

The protein autophagy related 7 (ATG7) is involved in the autophagic degradation of ferritin [41]. Therefore, we transfected cells with two independent siRNAs against ATG7 and examined cells for markers of ferroptosis. For both siRNAs, protein levels of ATG7 were reduced in U87MG, U251 and P3#GBM cells compared to controls (Supplementary Fig. 6a). Ferrous iron (Supplementary Fig. 6b), cell death (Supplementary Fig. 6c, 6d) and MDA (Supplementary Fig. 6e, 6f) were all decreased in U87MG- and P3#GBM-sh-COPZ1#1 cells transfected with si-ATG7#1 and si-ATG7#2 compared to the corresponding controls. These data demonstrate that depletion of COPZ1 induces ferroptosis in glioma cells by increasing NCOA4 and ATG7 levels. Thus, the COPZ1/NCOA4/FTH1 axis may be a novel therapeutic target in the treatment of gliomas.

### Downregulation of COPZ1 inhibits growth of GBM cells in vivo

**Fig. 7:**
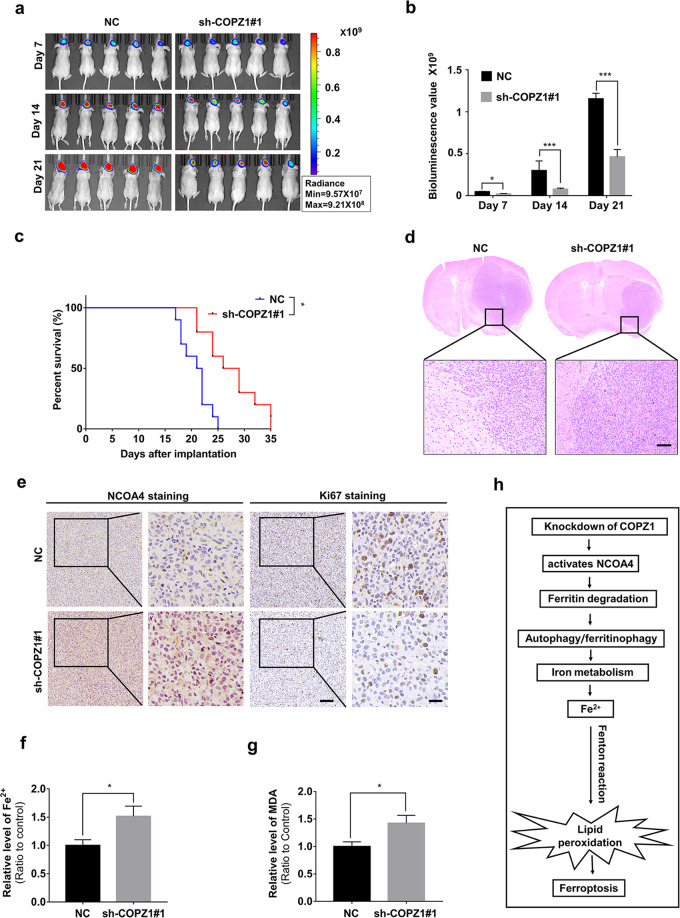
Down-regulation of COPZ1 reduces in vivo tumor growth. **a** Images of Intracranial tumor growth of luciferase expressing U87MG-sh-COPZ1#1 cells or U87MG-NC cells monitored at days 7, 14, and 21 after implantation using the IVIS-200 imaging system to detect bioluminescence. **b** Quantification of the bioluminescent signals from the orthotopic tumors in mice implanted with U87MG-sh-COPZ1#1 cells or U87MG-NC cells at days 7, 14, and 21. **c** Kaplan–Meier analysis of overall survival of tumor bearing animals. A log-rank test was used to assess the statistical significance of the differences. **d** Representative images of hematoxylin and eosin-stained sections from brains of orthotopic U87MG-sh-COPZ1#1 or U87MG-NC tumor bearing nude mice. Scale bar, 100 µm. **e** Representative images of immunohistochemical staining for NCOA4 and Ki67 in sections from brains of orthotopic U87MG-sh-COPZ1#1 or U87MG-NC tumor bearing nude mice. Scale bar third column, 100 μm; scale bar fourth column, 25 μm. **f** Comparison of ferrous iron levels in orthotopic U87MG-sh-COPZ1#1 or U87MG-NC xenograft samples. **g** Comparison of MDA levels in orthotopic U87MG-sh-COPZ1#1 or U87MG-NC xenograft samples. **h** Schematic figure of the COPZ1 induction of ferroptosis in GBM. Student’s *t* test for two-group comparison: *p < 0.05, **p < 0.01, ***p < 0.001; log-rank test: *p* < 0.05.

To determine the effect of COPZ1 on cell growth in vivo, we implanted luciferase expressing U87MG-sh-COPZ1#1 and U87MG-NC cells into the brains of nude mice (*n* = 10 mice in each group). Tumor growth was monitored at weekly intervals over 21 days using bioluminescence. Differences in bioluminescence values were significant between the two groups of animals by day 14 (Fig. 7a). At day 21 after implantation, the mean total flux was approximately 60% less in U87MG-sh-COPZ1#1 tumors than in the NC group (*p* < 0.001; Fig. 7b). Kaplan–Meier analysis of the survival data demonstrated that the overall survival of tumor bearing animals increased from 20.8 days (control group) to 27.8 days (knockdown group, *p* < 0.05; Fig. 7c). On histological examination, U87MG-sh-COPZ1#1 tumors were found to be smaller than NC tumors (Fig. 7d). Proliferation was also reduced based on immunostaining of the nuclear proliferation marker Ki67 on sections from U87MG-sh-COPZ1#1 tumors, while the expression of NCOA4 was increased (Fig. 7e). Furthermore, ferrous iron and MDA levels were increased in the U87MG-sh-COPZ1#1 tumors (Fig. 7f, g). Together, these findings demonstrated that loss of COPZ1 inhibited GBM tumor growth possibly through ferroptosis mediated tumor cell death.

## Discussion

A substantial amount of evidence obtained over the past 10 years indicates that changes in iron uptake and iron management are essential features of neoplastic cells [42]. Changes in iron metabolism are now considered a key metabolic “hallmark” of cancer [43]. Iron metabolic reprogramming and iron homeostasis dysfunction have also been shown to occur in GBM [44]. In this work, we analyzed genomic datasets for human glioma and discovered that high expression of COPZ1 was associated with poor prognosis and increasing tumor malignancy. COPZ1 expression was also upregulated in GBM cell lines compared to NHA. SiRNA knockdown of COPZ1 stimulated GBM cells in vitro to form autophagosomes and led to increased levels of the autophagy flux marker LC3B-II. Finally, COPZ1 shows a tendency to negatively regulate NCOA4 activity and knockdown of COPZ1 induces NCOA4-mediated ferritinophagy (Fig. 7h). This pathway thus might represent a novel approach, by induction of ferroptosis through loss of COPZ1, for treatment of human GBM.

The role of autophagy has been controversial, as it is thought to be activated as a mechanism of survival when tumor cells encounter stresses such as anticancer drugs, starvation and hypoxia [45, 46]. In this study, we found that the autophagic process involving ferritin, and ferritinophagy, caused GBM cell death via ferroptosis. We demonstrated that COPZ1 and NCOA4 colocalized in the cytoplasm, and that knockdown of COPZ1 increased ferritin protein levels in GBM cells. NCOA4 has been proven to be a hallmark of ferritinophagy, which plays a key role in degenerative diseases and cancers [47]. Our study further confirms a role for NCOA4 in GBM pathophysiology, which might help us further understand the pathogenesis of GBM and develop therapies based on the induction of ferroptois to treat cancer.

Studies show that it is difficult to inhibit GBM cells with chemical drugs alone [48]. This necessitates an urgent need to explore new therapeutic targets to improve the effectiveness of GBM treatment. Increasing iron levels in GBM cells in various ways to induce ferroptosis in tumor cells now appears to be an effective therapeutic approach [11, 49]. Our finding of the induction of the COPZ1-based ferroptosis signaling pathway in GBM provides a new possibility for treatment of this disease and possibly others, as COPZ1 also appears to play an important role in the occurrence and development of other types of solid tumors based on analysis of related databases.

Collectively, our data strongly suggest that autophagy plays an important role in regulating ferroptosis by increasing intracellular iron metabolism and cellular ROS accumulation. Knockdown of COPZ1 induces ferritinophagy and activates ferroptosis, as a result of the degradation of the intracellular iron storage protein ferritin through an NCOA4-mediated pathway. Ferrous iron levels are elevated, triggering the Fenton reaction which induces an increase in ROS. ROS causes lipid peroxidation, which leads to ferroptosis. Thus, the COPZ1/NCOA4/FTH1 axis and the iron upregulation demonstrated here may be a novel therapeutic target in the treatment of human GBM.

## Materials and methods

### Cell lines and cultures

Human glioma cell lines U87MG, U251, A172, LN229 and T98 were purchased from the Chinese Academy of Sciences Cell Bank (Shanghai, China). NHA and primary human GBM biopsy propagated tumor cells P3#GBM were kindly provided by Prof. Rolf Bjerkvig at the Department of Biomedicine, University of Bergen, Norway. Detailed protocols are provided in Supplementary “Materials and Methods”.

### SiRNA transfections

Gene-specific and negative control siRNAs were synthesized by GenePharma (Shanghai, China) and transfected into U87MG, U251 and P3#GBM cells for 48 h using Lipofectamine 2000 (Thermo Fisher Scientific) according to the manufacturer’s protocol. Detailed protocols are provided in Supplementary “Materials and Methods”.

### Immunohistochemistry

Blinded review of the images and staining was performed independently by two experienced neuropathologists (see Supplementary “Materials and Methods”). Staining of cancer cells within the sections was scored as follows: 0, no staining; 1, weak staining in <50% cells; 2, weak staining in ≥50% cells; 3, strong staining in <50% cells; and 4, strong staining in ≥50% cells.

### Western blot analysis

Cells and tissues were collected and lysed with RIPA lysis buffer (Thermo Fisher Scientific) supplemented with the proteinase inhibitor PMSF (Solarbio, Beijing, China) at a ratio of 100:1 (v/v). Protein concentration was determined with the BCA Protein Assay Kit (Beyotime). Equal quantities (20 ug) of protein extracts were separated with 10% SDS-PAGE and transferred to PVDF membranes (Merck Millipore; Billerica, MA, USA). The membrane was blocked with skimmed milk for 1 h and incubated with primary antibodies overnight at 4 °C.Antibodies are listed in Supplementary “Materials and Methods”. For detection, membranes were incubated with horseradish peroxidase-conjugated secondary antibodies (ZSGB-BIO) dissolved in antibody dilution buffer (Beyotime) for 1 h at room temperature. The membranes were visualized with chemiluminescence (Bio-Rad; Hercules, CA, USA) according to the manufacturer’s protocol.

### Iron assay

Ferrous iron concentration was analyzed in U87MG, U251 and P3#GBM cells using an iron colorimetric assay kit (Iron Assay Kit, Abcam; Burlingame, California, USA), Detailed protocols are provided in Supplementary “Materials and Methods”. The iron concentration was calculated according to the following formula: Iron concentration = (Sa/Sv) _*_ D. Sa: content of iron in the sample well calculated from the standard curve (nM), Sv: volume of sample added into the reaction wells (μL), D: sample dilution factor.

### Lipid peroxidation assessment

A MDA assay kit (Beyotime) was used to determine lipid peroxidation levels in U87MG, U251 and P3#GBM cells. A detailed protocols are provided in Supplementary “Materials and Methods”. The MDA content was expressed as a ratio of the absorbance value between treated cells and control cells.

### Animal studies

For intracranial xenograft studies, U87MG-NC and U87MG-sh-COPZ1#1 glioma cells were implanted into 4-week old female nude mice (*n* = 20; Shanghai SLAC Laboratory Animal Co., Shanghai, China). Detailed protocols are provided in Supplementary “Materials and Methods”.

### Statistical analysis

The Student’s *t* test for paired data was used to compare mean values. ANOVA was used to analyze potential differences between two groups with continuous variables. A two-sided *χ*^2^ -test was used to determine the association between *COPZ1* expression and clinicopathological features. Kaplan–Meier survival curves were compared using the log-rank test to assess survival differences between groups. Statistical analysis was conducted using GraphPad Prism version 7.00 software (GraphPad; La Jolla, CA, USA). All the experiments were repeated at least three times with triplicates unless stated otherwise. All tests were two-sided, and *P* values < 0.05 were considered to be statistically significant.

## Supplementary information

Supplemental material

Author agreement
