# Supplementary material for: Loss of COPZ1 induces NCOA4 mediated autophagy and ferroptosis in glioblastoma cell lines
Source: Oncogene. 2021 Jan 8;40(8):1425–39. doi: 10.1038/s41388-020-01622-3 (PMC7906905; doi:10.1038/s41388-020-01622-3)
Supplement: Supplementary file 1 — Supplemental material [file 41388_2020_1622_MOESM1_ESM.doc]

Supplementary information:

Loss of COPZ1 induces NCOA4 mediated autophagy and ferroptosis in glioblastoma cell lines

*Yulin Zhang^1,2,3^, Yang Kong^1,2,3^, Yuan Ma^1,2^, Shilei Ni^1,2^, Tobias Wikerholmen^3^, Kaiyan Xi^1,2^, Feihu Zhao^1,2^, Zhimin Zhao^1,2^, Junpeng Wang^1,2^, Bin Huang^1,2^, Anjing Chen^1,2^, Zhong Yao^1,2^, Mingzhi Han^3^, Zichao Feng^1,2^, Yaotian Hu^1,2^, Frits Thorsen^1,3,4*^, Jian Wang^1,2,3*^ and Xingang Li^1,2*^*

^1^ Department of Neurosurgery, Qilu Hospital and Institute of Brain and Brain-Inspired Science, Cheeloo College of Medicine, Shandong University, Shandong, 107 Wenhua Xi Road, Jinan, 250012, P.R. China

^2^ Shandong Key Laboratory of Brain Function Remodeling, Shandong, 107 Wenhua Xi Road, Jinan, 250012, P.R. China

^3^ Department of Biomedicine, University of Bergen, Jonas Lies vei 91, 5009 Bergen, Norway

^4^ Molecular Imaging Center, Department of Biomedicine, University of Bergen, Jonas Lies vei 91, 5009 Bergen, Norway

**Correspondence:**

Xingang Li, E-mail: lixg@sdu.edu.cn

Jian Wang, E-mail: Jian.Wang@uib.no

Frits Thorsen, E-mail: Frits.Thorsen@uib.no

**
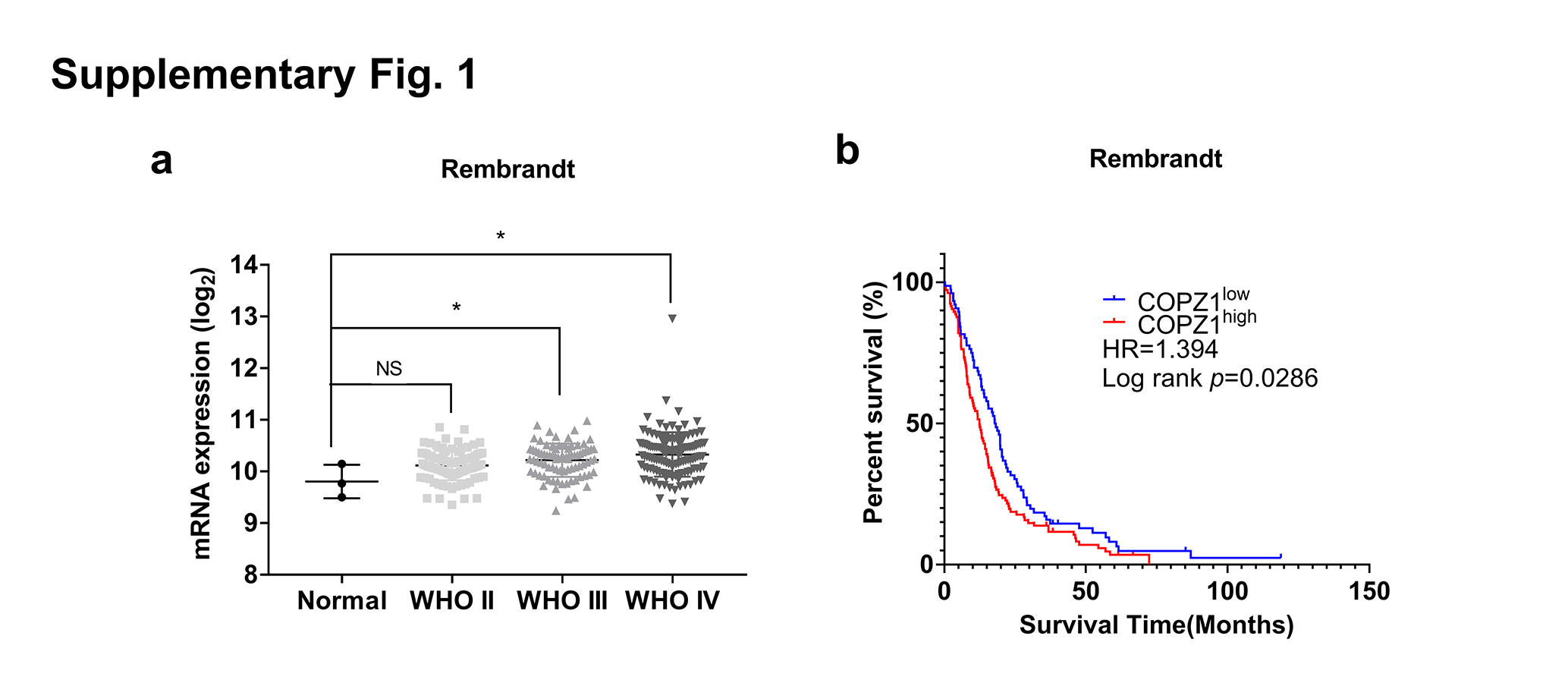
**

**Supplementary Figure. 1**

**(a)** *COPZ1* RNA expression (log_2_) from the Rembrandt database based on 2016 WHO classification. **(b)** Kaplan-Meier analysis showing overall survival of glioma patients based on the expression of *COPZ1* from the Rembrandt dataset. Data are shown as mean ± the standard error of the mean (SEM) for each group. One-way ANOVA for multi-group comparisons: NS = non-significant, ***p* < 0.01; log-rank test: *p* < 0.05.


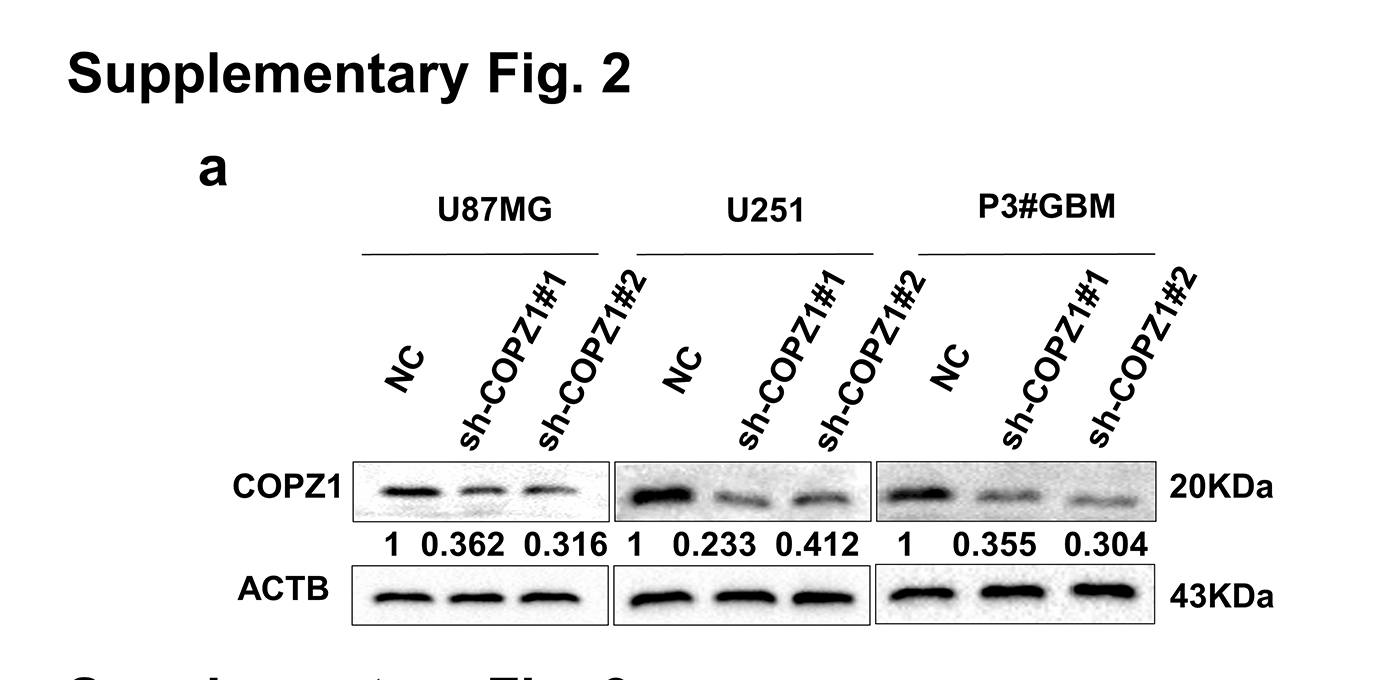


**Supplementary Figure 2**

**(a)** Western blot analysis of COPZ1 protein levels in U87MG, U251 and P3#GBM cells infected with lentiviral constructs expressing sh-COPZ1#1 and sh-COPZ1#2.

**
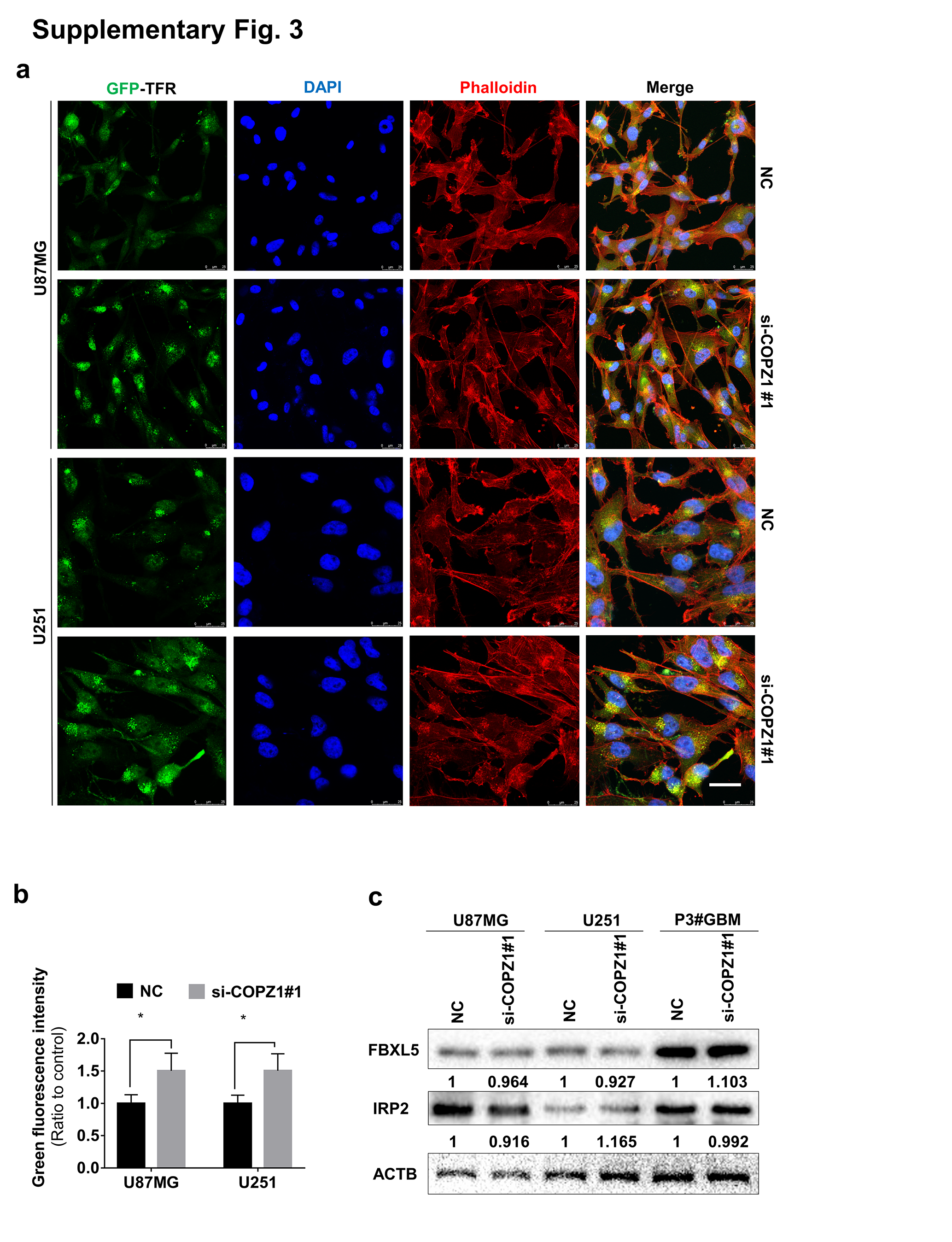
**

**Supplementary Figure 3**

**(a)** Representative fluorescence images to detect TFR protein in U87MG and U251 cells. DAPI was used to stain [cell](../../../../C:/Users/Robin/AppData/Local/youdao/dict/Application/8.8.0.0/resultui/html/index.html#/javascript:;) nuclei; phalloidin was used to stain F-actin to visualize the distribution of microfilaments in the cytoskeleton of cells. Scale bars, 25 μm. **(b)** Statistical analysis of the images shown in **(a)**. **(c)** Western blot analysis of FBXL5 and IRP2 protein levels in U87MG, U251 and P3#GBM cells infected with si-COPZ1. Student’s *t*-test for two-group comparison: **p* < 0.05, ***p* < 0.01, ****p* < 0.001.

**
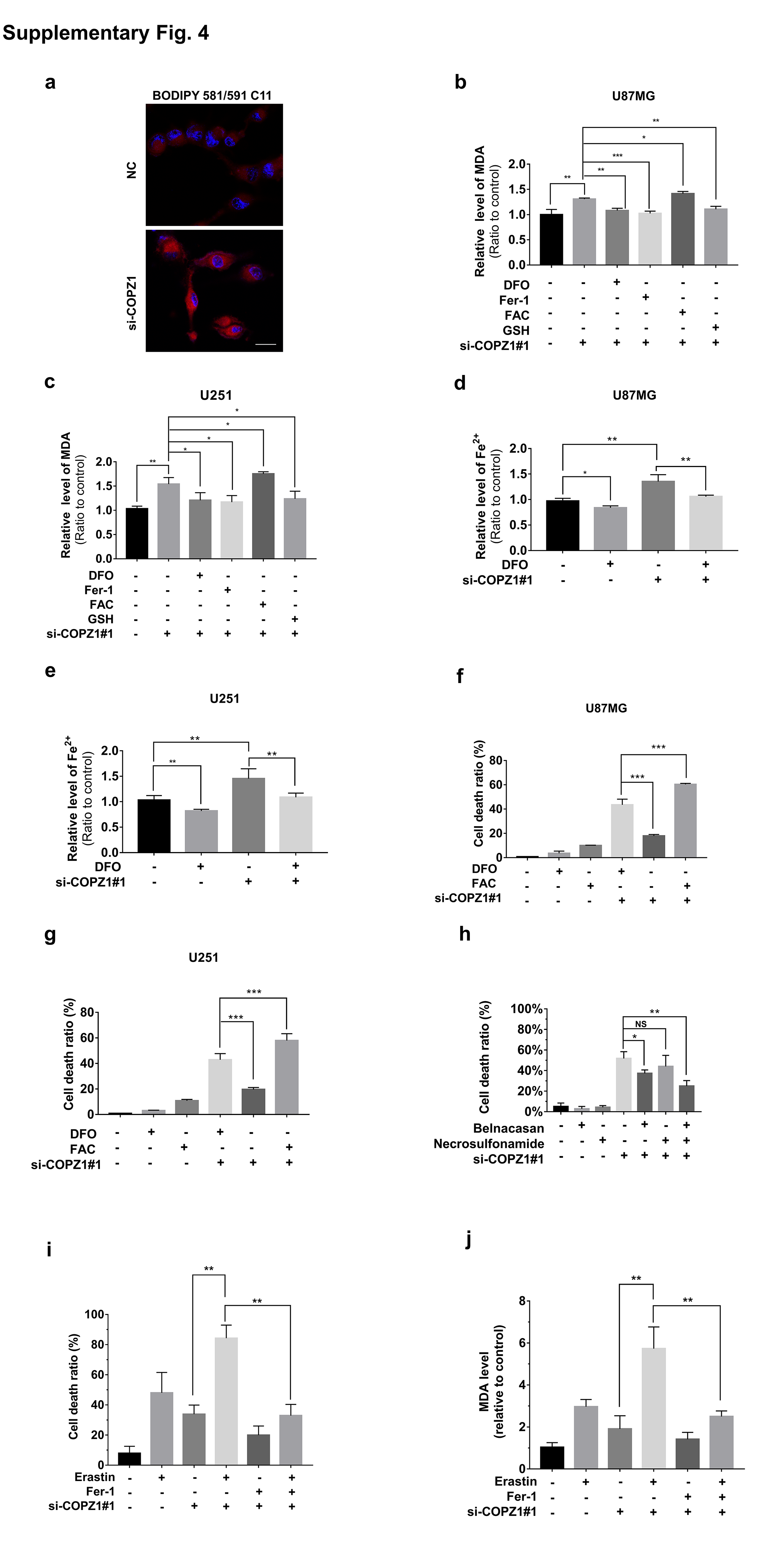
**

**Supplementary Figure 4**

**(a)** Representative images of BODIPY 581/591 C11 (red) staining in U87MG cells. DAPI was used to stain [cell](../../../../C:/Users/Robin/AppData/Local/youdao/dict/Application/8.8.0.0/resultui/html/index.html#/javascript:;) nuclei. Scale bars, 25 μm. **(b)** MDA levels in si-COPZ1#1 transfected U87MG cells pretreated with DFO, Fer-1, FAC or GSH. **(c)** MDA levels in si-COPZ1#1 transfected U251 cells pretreated with DFO, Fer-1, FAC or GSH. **(d)** Ferrous iron levels in si-COPZ1#1 transfected U87MG cells pretreated with DFO detected with an iron assay kit. **(e)** Ferrous iron levels in si-COPZ1#1 transfected U251 cell lines pretreated with DFO detected with an iron assay kit. **(f)** LDH release assay to detect cell death in si-COPZ1#1 transfected U87MG cells pretreated with DFO or FAC. **(g)** LDH release assay to detect cell death in si-COPZ1#1 transfected U251 cells pretreated with DFO or FAC. **(h)** Cell death ratio of U867MG cells treated with belnacasan and/or necrosulofonamide. **(i)** Cell death ratio in si-COPZ1#1 transfected U87MG cells treated with erastin or/and Fer-1. **(j)** MDA levels in si-COPZ1#1 transfected U87MG cells treated with erastin or/and Fer-1. One-way ANOVA for multi-group comparisons: NS = non-significant, **p* < 0.05, ***p* < 0.01, ****p* < 0.001.

**
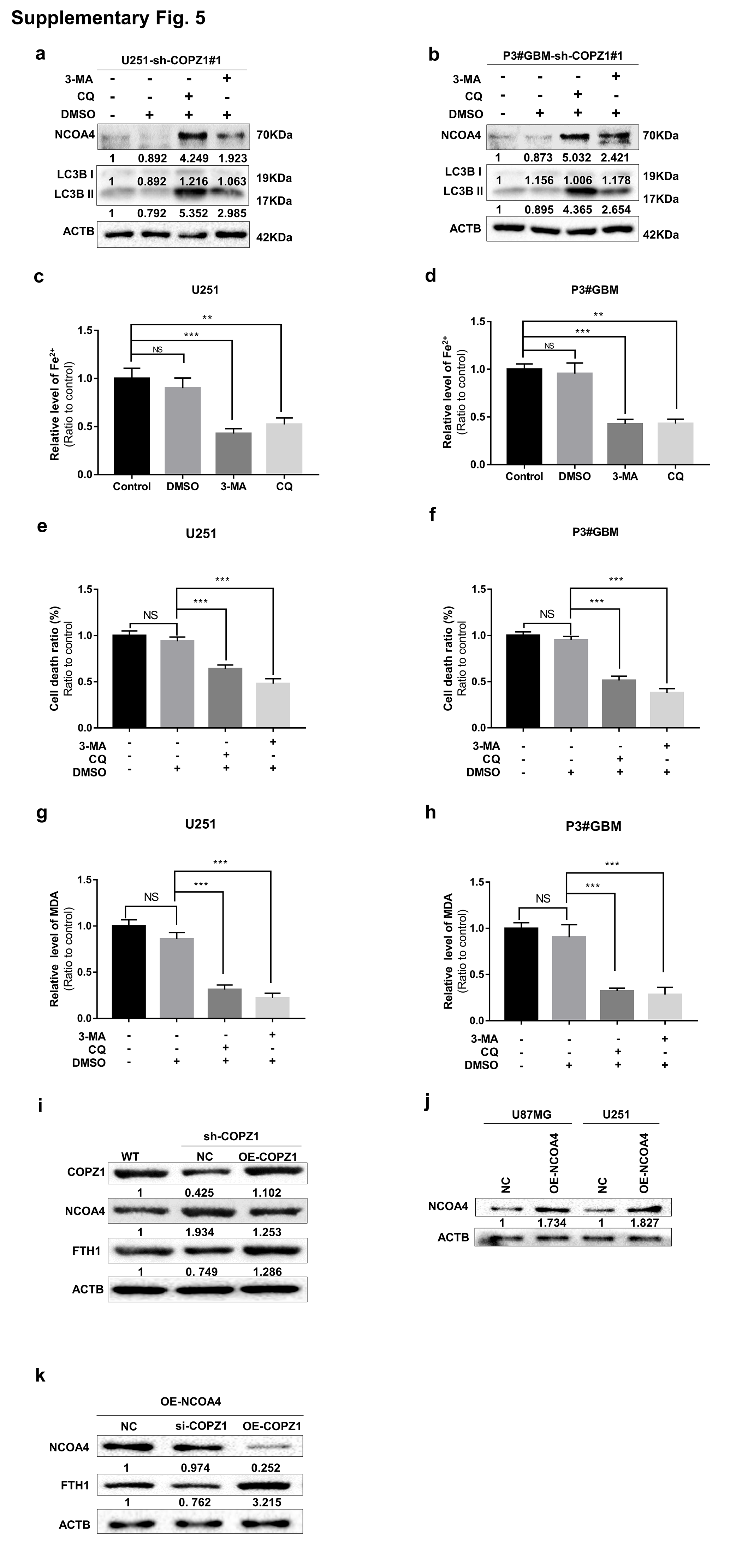
**

**Supplementary Figure 5**

**(a)** Western blots showing levels of NCOA4 and LC3B in U87MG-sh-COPZ1#1 cells after pretreatment with 3-MA (10 mM) and CQ (3 μM) for 1 h. **(b)** Western blots showing levels of NCOA4 and LC3B in P3#GBM-sh-COPZ1#1 cells after pretreatment with 3-MA (10 mM) and CQ (3 μM) for 1 h. Pretreatment of cells with 3-MA (10 mM) or CQ (3 μM) for 1 h and determination of ferrous iron levels in **(c)** U251-sh-COPZ1#1 and **(d)** P3#GBM-sh-COPZ1#1 cells; cell death ratio in **(e)** U251-sh-COPZ1#1 and **(f)** P3#GBM-sh-COPZ1#1 cells; and MDA levels in **(g)** U251-sh-COPZ1#1 and **(h)** P3#GBM-sh-COPZ1#1 cells. **(i)** Western blots showing rescue experiments with overexpression of COPZ1. **(j)** Western blot analysis of NCOA4 protein levels in U87MG and U251 cells infected with OE-NCOA4. **(K)** Western blot analysis of NCOA4 and FTH1 protein levels in NCOA4 overexpressed U87MG cells. One-way ANOVA for multi-group comparisons: NS = non-significant, **p* < 0.05, ***p* < 0.01, ****p* < 0.001.

**
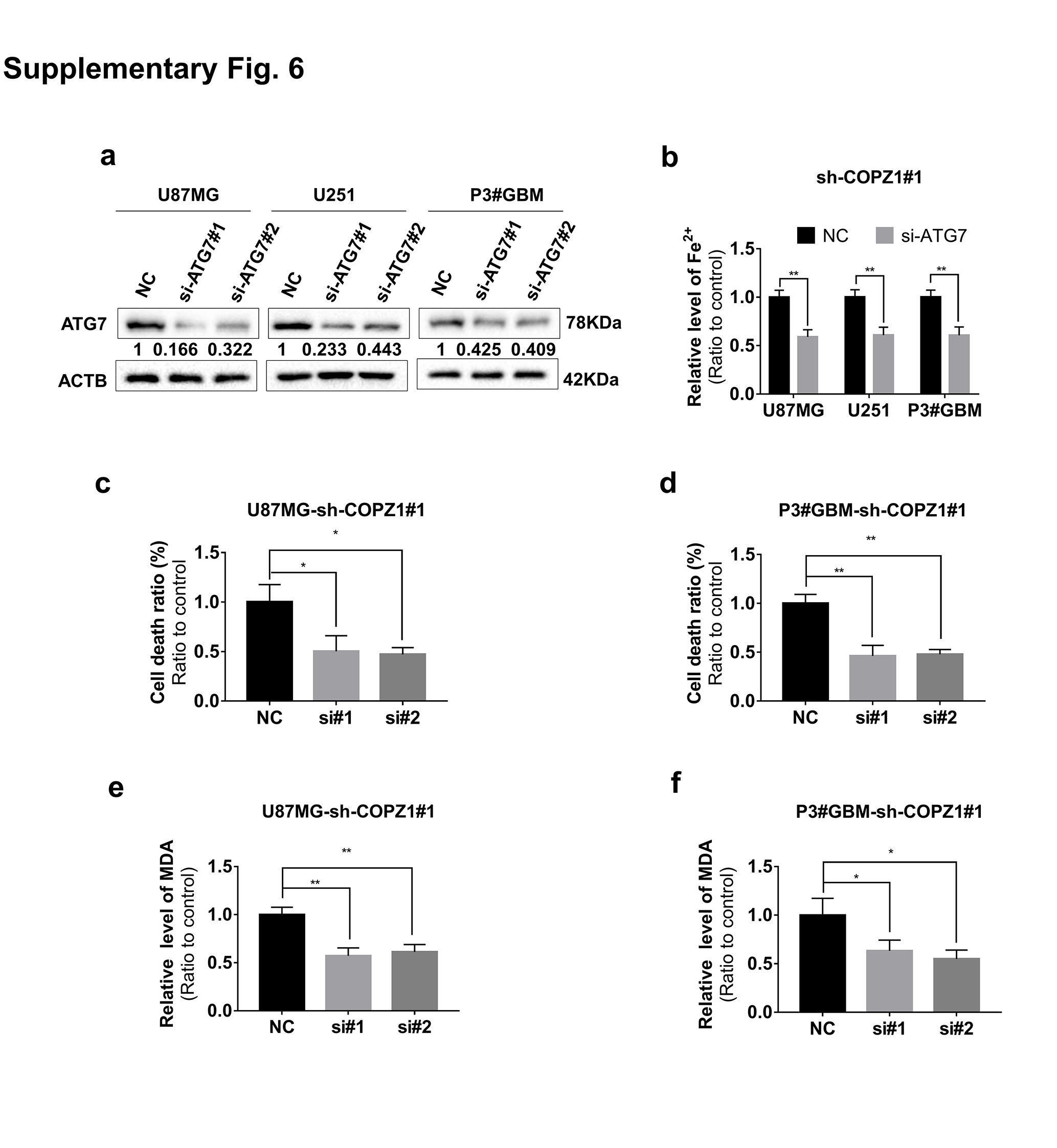
**

**Supplementary Figure 6**

**(a)** Western blots showing levels of ATG7 and ACTB (loading control) in U87MG, U251 and P3#GBM glioma cells transfected with si-ATG7#1 and si-ATG7#2 for 48 h. (b) Ferrous iron levels detected in U87MG-, U251- and P3#GBM-sh-COPZ1#1 cells transfected with ATG7 siRNAs. (c) The effects of knockdown of ATG7 by siRNAs on cell viability in U87MG-sh-COPZ1#1 cells. (d) LDH release assay to detect cell death in P3#GBM-sh-COPZ1#1 cells transfected with ATG7 siRNAs. (e) MDA levels in U87MG-sh-COPZ1#1 cells transfected with ATG7 siRNAs. (F) MDA levels in P3#GBM-sh-COPZ1#1 transfected with ATG7 siRNAs. Student’s t-test for two-group comparison: **p < 0.01; one-way ANOVA for multi-group comparisons: NS = non-significant, *p < 0.05, **p < 0.01, ***p < 0.001.

**Supplementary Materials and Methods**

**Cell culture**

U87MG, U251, A172, LN229 and T98 cells were cultured in Dulbecco’s modified Eagle’s medium (DMEM; Thermo Fisher Scientific; Waltham, MA, USA) supplemented with 10% fetal bovine serum (FBS; Thermo Fisher Scientific) in 5% CO_2_ in a humidified incubator at 37°C. P3#GBM cells were cultured in Neurobasal^TM^ Medium (Gibco, Thermo Fisher Scientific) and supplemented with 2% B27 Neuro Mix (Thermo Fisher Scientific), 20 ng/mL epidermal growth factor (EGF; Thermo Fisher Scientific), and 10 ng/mL basic fibroblast growth factor (bFGF; PeproTech; Rocky Hill, NJ, USA) in 5% CO_2_ in a humidified incubator at 37°C. Accutase (Thermo Fisher Scientific) was used to digest tumor spheres for expansion of GSCs.

**SiRNA transfections**

Cells were seeded at a density of 3 x 10^5^ cells/well in 6-well plates. The siRNA duplexes were transfected into cells when the cells were 70-80% confluent. The following siRNA sequences were used to target the RNAs indicated: COPZ1 #1 and COPZ1 #2, 5’-CCAUCGGACUGACAGUGAAATT-3’ and 5’- CCGGCCTGTATACTGTCAAAGCCAT-3’; NCOA4 #1 and NCOA4 #2, 5’-ACTCTTGTTTATCGAAGT

ATA-3’ and 5’-CTCTTATTCCAGTCCTATAAT-3’; ATG7 #1 and ATG7 #2, 5’-GGAGTCACAGCTCTTCC

TT-3’ and 5’-CAGCTATTGGAACACTGTA-3’. Western blotting was used to evaluate siRNA knockdown efficiency.

**ShRNA transfections**

Short hairpins sh-COPZ1#1 and sh-COPZ1#2 (5’-CCAUCGGACUGACAGUGAAATT-3’ and 5’- CCGGCCTGTATACTGTCAAAGCCAT-3’) were ligated to the lentiviral vector pLKO.1 containing a puromycin resistant region (GenePharma). Luciferase-expressing U87MG, U251 and P3#GBM cells were infected with the shRNA lentiviruses. After 48 h, the medium was removed and replaced with fresh medium containing 2 μg/mL puromycin (Thermo Fisher Scientific) for selection for an additional 2 weeks to enrich for cells harboring the constructs. Western blotting was performed to assess shRNA knockdown efficiency, and cells were split for different assays.

**Cell viability and proliferation assays**

Cell viability in U87MG, U251 and P3#GBM cells was assessed using the Cell Counting Kit-8 assay (CCK-8) (Dojindo; Kumamoto, Japan) according to the manufacturer’s protocol. Cells were seeded at 2×10^3^ cells/well in 96-well plates and incubated at 37°C for 24, 48 or 72 h in a humidified chamber containing 5% CO_2_. CCK-8 solution (10 μL) was added to each well, and the plates were incubated for 1 h at 37°C in the incubator. The optical absorbance in each well was read at 450 nm (OD450) using a microplate reader (Bio-Rad; Hercules, CA, USA). Proliferation was assessed using the EdU incorporation assay according to the manufacturer’s protocol (Rib-bio; Guangzhou, China). Briefly, EdU was incorporated into proliferating cells and detected through a catalyzed reaction with a fluorescently labeled azide. Labeled cells were examined under fluorescence microscopy, and the number of EdU-positive cells was counted from 500 cells in three independent experiments.

**Immunohistochemistry**

Tissue samples were fixed in 4% paraformaldehyde in phosphate buffered saline (PBS) and embedded in paraffin. Sections (4 µm) were de-waxed, rehydrated, and incubated in 0.01 M citrate buffer for 20 min at 95°C for antigen retrieval. Endogenous peroxidase activity was blocked with 3% hydrogen peroxide (ZSGB-Bio; Beijing, China) and non-specific antigens were blocked with 10% normal goat serum (ZSGB-Bio), followed by incubation with primary antibody at 4°C for 12 h. The following primary antibodies were used: rabbit anti-COPZ1 (20440-1-AP, 1:100; Proteintech, Wuhan, China) and rabbit anti-Ki67 (ab15580, 1:500; Abcam, Cambridge, UK). Sections were rinsed with PBS (3 x for 5 min) and incubated with goat anti-rabbit secondary antibody (ZSGB-Bio). The antigens were visualized using the hydrogen peroxidase substrate 3,3’-diaminobenzidine (DAB, ZSGB-Bio), and the slides were counterstained with hematoxylin (Beyotime; Shanghai, China) at 25°C for 2 min. For negative controls, sections were incubated with normal goat serum rather than primary antibody.

**Antibodies**

The following primary antibodies were used: rabbit anti-beta actin (ab8226,1:2000, Abcam), rabbit anti-COPZ1 (20440-1-AP, 1:1000, Proteintech; Rosemont, IL, USA), rabbit anti-NCOA4 (ab86707, 1:2000, Abcam), rabbit anti-ferritin heavy chain (ab75972, 1:1000, Abcam), rabbit anti-LC3B (ab192890,1:2000), rabbit anti-SQSTM1/p62 (ab109012, 1:10000, Abcam), rabbit anti-Atg7 (#8558, 1:1000, Cell Signaling Technology; Waltham, MA, USA).

**Iron assay**

Briefly, cells were added to iron assay buffer on ice and centrifuged at 16,000g for 10 min at 4°C to obtain the supernatant. 50 μL of the supernatant was incubated with 50 μL of assay buffer in a 96 multi-well microplate for 30 min at 25°C. Samples were incubated with 100 μL of the iron probe for 60 min at 25°C while protected from light. The absorbance at 593 nm was measured using a microplate reader (Bio-Rad). The experiments were performed in triplicate.

**Live/dead staining**

The numbers of live and dead cells were assessed using a Live/Dead Viability/Cytotoxicity Kit (Sigma-Aldrich; St. Louis, MO, USA), according to manufacturer’s protocol. The working solutions with calcein-AM and ethidium homodimer-1 were prepared in PBS at a proper dilution. The staining solutions were mixed into a working solution with the medium at a ratio of 1:2 (v/v) and incubated at 37°C for 15 min. Images of live and dead cells were captured under confocal microscopy (Leica SP8 confocal microscope, Leica Microsystems; Wetzlar, Germany).

**Lactate dehydrogenase release cell death assay**

Cell death ratios in U87MG, U251 and P3#GBM cells were detected using the Lactate Dehydrogenase Cytotoxicity Assay Kit (Beyotime), according to the manufacturer's instructions. After treatment, the 96-well cell culture plate was centrifuged at 400g for 5 min and the supernatant was removed. The LDH release reagent provided in the kit (150 μL; diluted 1:10 with PBS) was added to each well, and plates were incubated at 37°C for 1 h. The absorbance at 490 nm (OD490) was measured using a microplate reader (Bio-Rad). All experiments were performed in triplicate. The results were calculated according to the following formula: Cell death ratio % = (absorbance of processed sample - absorbance of sample control well) / (absorbance of cell maximum enzyme activity - absorbance of sample control well) × 100.

**Superoxide anion detection**

The levels of superoxide anion in U87MG, U251 and P3#GBM cells were detected using dihydroethidium (DHE, Beyotime). Cells were cultured in µ-Slide 8 well chamber slides (Ibidi; Martinsried, Germany) for 24 h, rinsed three times with PBS and then loaded with DHE (10 mmol/L) in fresh medium at 37°C for 30 min. Fluorescence was measured at an excitation wavelength of 485 nm and an emission wavelength of 530 nm using a Leica SP8 [confocal](#/javascript:;) [microscope](#/javascript:;) (Leica Microsystems; Wetzlar, Germany). The levels of superoxide anions were expressed as a ratio of the absorbance values between treated and control cells. The fluorescence intensity was calculated using ImageJ software (https://imagej.nih.gov/ij/). For each group, three representative images from experiments performed in triplicate were chosen for statistical analysis.

**Mitochondrial membrane potential assay**

Mitochondrial membrane potential in U87MG and U251 cells was detected using the JC-1 Assay Kit (Beyotime), according to the manufacturer’s protocol. Cells were stained with JC-1 working solution for 20 min at 37°C in an incubator and analyzed using confocal microscopy (Leica SP8 [confocal](#/javascript:;) [microscope](#/javascript:;); Leica Microsystems). The intensities of red (excitation: 530 nm; emission: 590 nm) and green fluorescence (excitation: 485 nm; emission: 528 nm) were measured. Assays were performed in triplicate, and fluorescence intensities were calculated using ImageJ software.

**Lipid peroxidation assessment**

Cells were added to lysis buffer on ice, homogenized, and centrifuged at 1600g for 10 min at 4°C to collect the supernatant for analysis according to the manufacturer’s protocol. 100 μL of the supernatant was incubated with 100 μL of the test solution for 15 min at 100°C in 96 multi-well plate, and then cooled down to room temperature. The mixture was centrifuged at 1000g for 10 min to obtain the supernatant, and the absorbance was read at 530 nm using a microplate reader (Bio-Rad).

**BODIPY581/591 staining**

To visualize the lipid peroxidation, U87MG cells were seeded in 8-well ibidi plates and transfected with si-COPZ1#1. After 48 h, cells were stained with 2 μmol/L C11-BODIPY581/591 probe (Thermo Fisher Scientific) according to the manufacturer's instructions. After 30 min of incubation at 37°C in the dark, the cells were stained with Hoechst (Beyotime) for 10 min. Then, cells were washed with PBS solution and observed using a Leica SP8 confocal laser scanning microscope.

**Animal studies**

Mice were divided into two groups (10 mice per group) and anesthetized with an intraperitoneal injection (80 μL) containing ketamine HCl (25 mg/mL), xylazine (2.5 mg/mL), and 14.25% ethyl alcohol (diluted 1:3 in 0.9% NaCl). U87MG-NC and U87MG-sh-COPZ1#1 glioma cells (10^6^ cells diluted in 10 μL PBS per animal) were injected into the right frontal lobes of each mouse using the following coordinates: 1 mm anterior and 2.5 mm lateral to the bregma, at a depth of 2 mm. Animals displaying symptoms, such as severe hunchback posture, apathy, decreased motion or activity, dragging legs, unkempt fur, or drastic loss of body weight, were [sacrifice](#/javascript:;)d by cervical dislocation. The mice were perfused with physiological saline and 4% paraformaldehyde (PFA). The brains were harvested, ﬁxed in 4% PFA, embedded in parafﬁn and sectioned (4 µm) for H&E and IHC staining.
