# Supplementary material for: Loss of COPZ1 induces NCOA4 mediated autophagy and ferroptosis in glioblastoma cell lines
Source: Oncogene. 2021 Jan 8;40(8):1425–39. doi: 10.1038/s41388-020-01622-3 (PMC7906905; doi:10.1038/s41388-020-01622-3)
Supplement: Supplementary file 2 — Author agreement [file 41388_2020_1622_MOESM2_ESM.pdf]

Author agreement

已发送 (无主题) × Re: Author... × Authorshi... ×

召回邮件 回复 回复全部 转发 移动到 标记为 更多 删除

Authorship agreement

发起会议
2020-12-02 13:53:21

发件人： "Xin-Gang Li" <lixg@sdu.edu.cn>

收件人： "jian.wang" <jian.wang@uib.no> frits.thorsen@uib.no yulindrive@163.com kongyang124817344@126.com 1015118285@qq.com nishilei@sdu.edu.cn tobias.wikerholmen@student.uib.no 562026423@qq.com zhminzhao@163.com zfhjn@163.com wang_junpeng@outlook.com hb@sdu.edu.cn chenaj@sdu.edu.cn eyaozhong@sina.com mingzhi.han@uib.no "冯子超" <fengzichao@mail.sdu.edu.cn> king_d_arthur@163.com zhwjsdu@163.com [↑隐藏信息]

附　件： 2 个(New Manuscript.doc 等...) 查看附件

▸ 对方已阅读 [查看详情] 共发给18个收件人，其中 15个成功到达对方服务器，3个信件已被对方阅读

Dear co-authors,

Our manuscript entitled "Loss of COPZ1 induces NCOA4 mediated autophagy and ferroptosis in glioblastoma cell lines" has been provisionally accepted for publication in Oncogene.

As we've discussed, we added new co-authors Feihu Zhao and Zhimin Zhao who have contributed during the revision process, and Wenjing Zhou gave up her authorship due to a change in work.

An agreement from all authors including additions and deletions is needed for further process our manuscript. **Please reply to this email to confirm your agreement to these changes.** Both the most recent and the original manuscripts are attached for your reference.

Best regards,

Xingang Li

普通附件(2 个) 全部下载 全部保存到个人网盘

New Manuscript.doc (142.00K)
下载 打开 预览 保存到个人网盘

Original Manuscript.doc (143.00K)
下载 打开 预览 保存到个人网盘

快捷回复给所有人

Re: Authorship agreement

发起会议
2020-12-02 14:05:58

发件人： "张玉霖" <yulindrive@163.com>

收件人： "Xin-Gang Li" <lixg@sdu.edu.cn>

Dear Xingang Li,

I agree with the changes.

Best,

Yulin Zhang

Re: Authorship agreement

发起会议
2020-12-02 14:13:13

发件人： "MY" <1015118285@qq.com>

收件人： "Xin-Gang Li" <lixg@sdu.edu.cn>

Dear Xingang Li,

I agree with the changes in the new manuscript regarding authorship.

Best,

Ma Yuan

Re:Authorship agreement

发起会议
2020-12-02 14:14:21

发件人： "zfhjn" <zfhjn@163.com>

收件人： "Xin-Gang Li" <lixg@sdu.edu.cn>

Dear Xingang Li,

I agree with the changes in the new manuscript regarding authorship.

Best,

Zhao Feihu

发自我的小米手机
在 Xin-Gang Li <lixg@sdu.edu.cn>，2020年12月2日 13:53写道：

Re:Authorship agreement

发起会议
2020-12-02 14:15:22

发件人： "冯子超" <fengzichao@mail.sdu.edu.cn>

收件人： "Xin-Gang Li" <lixg@sdu.edu.cn>

Dear Xingang Li,

I agree with the changes in the new manuscript regarding authorship.

Best regards,

Zichao Feng

回复：Authorship agreement

发起会议
2020-12-02 14:16:11

发件人： "chenaj@sdu.edu.cn" <chenaj@sdu.edu.cn>

收件人： "Xin-Gang Li" <lixg@sdu.edu.cn>

Dear Xingang Li,

I agree with the changes in the new manuscript regarding authorship.

Best,
Anjing Chen

发自我的华为手机

回复：Authorship agreement

发件人： "席开颜" <xkyzoe@qq.com>

收件人： "Xin-Gang Li" <lixg@sdu.edu.cn>

2020-12-02 14:16:58

发起会议

Dear Xingang Li， I agree with the changes in the new manuscript regarding authorship. Best， Kaiyan Xi

------------------ 原始邮件 ------------------

Re: Authorship agreement

发件人： "kongyang" <kongyang124817344@126.com>

收件人： "Xin-Gang Li" <lixg@sdu.edu.cn>

2020-12-02 14:18:00

发起会议

Dear Xingang Li,

I agree with the changes in the new manuscript regarding authorship.

Best,

Yang Kong

Re: Authorship agreement

发件人： "胡耀天" <king_d_arthur@163.com>

收件人： "Xin-Gang Li" <lixg@sdu.edu.cn>

2020-12-02 14:28:56

发起会议

Dear Xingang Li,

I agree with the changes in the new manuscript regarding authorship.

Best,

Yaotian Hu

Re:Authorship agreement

发件人： "赵志敏" <zhminzhao@163.com>

收件人： "Xin-Gang Li" <lixg@sdu.edu.cn>

2020-12-02 14:31:09

发起会议

Dear Xingang Li,

I agree with the changes in the new manuscript regarding authorship.

Best,

Zhimin Zhao

Re: Authorship agreement

发件人： "倪石磊" <nishilei@sdu.edu.cn>

收件人： "Xin-Gang Li" <lixg@sdu.edu.cn>

2020-12-02 14:32:59

发起会议

Dear Xingang Li,

I agree with the changes in the new manuscript regarding authorship.

Best,

Shilei Ni

Re: Authorship agreement

发起会议
2020-12-02 14:41:07

发件人： "Wenjing Zhou" <zhwjsdu@163.com>

收件人： "Xin-Gang Li" <lixg@sdu.edu.cn>

Dear Xingang Li,

I agree with the changes in the new manuscript regarding authorship.

Best,
Wenjing Zhou

发自我的iPhone

Re: Authorship agreement

发起会议
2020-12-02 15:03:55

发件人： hb@sdu.edu.cn

收件人： "Xin-Gang Li" <lixg@sdu.edu.cn>

Dear Xingang Li,

I agree with the changes in the new manuscript regarding authorship.

Best,

Bin Huang

Qilu Hospital of Shandong University

Re: Authorship agreement

发起会议
2020-12-02 15:28:44

发件人： "Wang Junpeng" <Wang_Junpeng@outlook.com>

收件人： "Xin-Gang Li" <lixg@sdu.edu.cn>

Dear Xingang Li,

I agree with the changes in the new manuscript regarding authorship.

Best,

Junpeng Wang

Svar: Authorship agreement

发起会议
2020-12-02 15:53:50

发件人： "Frits Alan Thorsen" <Frits.Thorsen@uib.no>

收件人： "Xin-Gang Li" <lixg@sdu.edu.cn> "Jian Wang" <Jian.Wang@uib.no> "yulindrive@163.com" <yulindrive@163.com> "kongyang124817344@126.com" <kongyang124817344@126.com> "1015118285@qq.com" <1015118285@qq.com> .. [↓还有13个联系人]

Dear Prof Xingang Li;

I agree to the changes made in the author list.

Best regards

Frits Thorsen

快捷回复给所有人

Re: Authorship agreement

发件人： "Mingzhi Han" <Mingzhi.Han@uib.no>
收件人： "Xin-Gang Li" <lixg@sdu.edu.cn>

发起会议
2020-12-02 16:55:46

Dear Xingang Li,

I agree with the changes in the new manuscript regarding authorship.

Best,
Mingzhi Han

Re: Authorship agreement

发件人： "Jian Wang" <Jian.Wang@uib.no>
收件人： "Xin-Gang Li" <lixg@sdu.edu.cn>
抄 送： "Frits Alan Thorsen" <Frits.Thorsen@uib.no> "张玉霖" <yulindrive@163.com> "kongyang" <kongyang124817344@126.com> "1015118285@qq.com" <1015118285@qq.com> "nishilei@sdu.edu.cn" <nishilei@sdu.edu.cn> .. [↓还有12个联系人]

发起会议
2020-12-02 16:57:23

Dear Professor Li:

I agree to the changes made in the author list.

With best regards

Jian Wang

发件人： "eyaozhong@sina.com" <eyaozhong@sina.com>
收件人： "Xin-Gang Li" <lixg@sdu.edu.cn>

发起会议
2020-12-02 17:54:52

Dear Xingang Li,

I agree with the changes in the new manuscript regarding authorship.

Best regard.

Zhong Yao

Re: Authorship agreement

发件人： "Tobias Espedal Wikerholmen" <Tobias.Wikerholmen@student.uib.no>
收件人： "Xin-Gang Li" <lixg@sdu.edu.cn>

发起会议
2020-12-03 17:28:03

Regarding the manuscript ""Loss of COPZ1 induces NCOA4 mediated autophagy and ferroptosis in glioblastoma cell lines". I hereby confirm that I agree to the beforementioned authorship changes, adding Feihu Zhao and Zhimin Zhao, and removing Wenjing Zhou.

Regards,
Tobias Wikerholmen
